# Novel Method to Measure Volumes of Retinal Specific Entities

**DOI:** 10.3390/jcm13164620

**Published:** 2024-08-07

**Authors:** Myrta Lippera, Mariantonia Ferrara, Karina Spiess, Nouf Alnafisee, Naseer Ally, Assad Jalil, Tsveta Ivanova, George Moussa

**Affiliations:** 1 Manchester Royal Eye Hospital, Manchester University Hospitals NHS Foundation Trust, Manchester M13 9WL, UK; 2 School of Medicine, University of Malaga, 29071 Malaga, Spain; 3 Department of Medical and Surgical Specialties, Radiological Sciences and Public Health, University of Brescia, 25123 Brescia, Italy; 4 Eye Unit, ASST Spedali Civili di Brescia, Piazzale Spedali Civili, 25123 Brescia, Italy; 5 Division of Ophthalmology, Department of Neurosciences, School of Clinical Medicine, Faculty of Health Sciences, University of the Witwatersrand, 7 York Road, Parktown, Johannesburg 2193, South Africa

**Keywords:** retinal volume, Spectralis, optical coherence tomography, volumetric analysis, retina

## Abstract

**Objectives:** The aim of this study is to describe and validate an optical-coherence-tomography (OCT)-based method to easily calculate specific volumes, addressing the limitations of current OCT software in automating volumetric analysis for specific entities in retinal pathologies. **Methods**: After manually drawing the specific entity on linear OCT scans using the calliper function and automated measurement of its area, the following formula was used for volumetric calculation: Volume [mm^3^] = ∑area [mm^2^] × OCT-scan distance [mm]. Retinal volume (RV) was measured by two independent observers in eyes with a normal foveal profile (NFP) and was compared with the automated measurements performed by the OCT software (Engineering GmbH, Heidelberg, Germany); the same process was repeated for the volume of the foveal cavity (FC) or foveoschisis (FS) in eyes with lamellar macular holes (LMHs). Power calculations were conducted to ensure adequate sample size. The measurements were re-acquired after six weeks. Intra- and inter-observer variability as well as comparison to automated RV calculations were analysed. **Results**: This study included a total of 62 eyes divided into two groups: the NFP (30 eyes) and LMH (32 eyes) groups. The Bland–Altman plots showed a high degree of agreement in both groups for inter-observer and intra-observer agreement. In addition, in the NFP group, a high degree of agreement was demonstrated between human observers and the OCT software (Spectralis). **Conclusions**: An easy, reliable, and widely applicable method to calculate volumes is described and validated in this paper, showing excellent inter- and intra-observer agreement, which can be applied to any entity requiring a specific study in the context of retinal pathologies.

## 1. Introduction

Diseases of the vitreoretinal interface result in a three-dimensional (3D) re-modelling of retinal tissue and there has been a growing interest recently in the potential role of retinal volumetric analysis in the diagnosis, treatment planning, and monitoring of these pathological entities [1,2,3,4,5,6,7,8]. Indeed, it has been suggested that retinal volumetric analysis may better reflect the in vivo alterations of retinal tissue caused by specific pathologies [1]. The clinical relevance of specific retinal volumes as biomarkers has already been demonstrated in several medical retinal diseases, such as diabetic retinopathy (DR), age-related macular degeneration (AMD), or glaucoma, ultimately contributing to improved patient outcomes and vision preservation [3,5,7,9,10,11]. In the vitreoretinal field, it was shown that the volume of specific entities, such as the foveal cavity (FC) in lamellar macular holes (LMHs), is associated with visual acuity at baseline and with the natural history of the disease during the follow-up period [1]. Moreover, in eyes with epiretinal membrane (ERM), higher pre-operative macular volumes were associated with worse visual outcomes after surgery [12].

Optical-coherence-tomography (OCT) uses light waves to acquire a reflectivity profile of the retina, showing cross-sectional images of in vivo 3D tissue structures [13]. Thanks to its fast acquisition, high-resolution, and non-invasive properties, OCT is an essential tool in ophthalmology for the diagnosis and monitoring of pathologies as well as for research purposes [14]. Several innovations aimed at improving its quality have been recently made, including the development of new automated analysis algorithms to detect retinal surfaces or layers allowing volumetric analysis [15,16]. Nevertheless, automated volumetric calculations are still limited to the total retinal volume and the volumes of single retinal layers; however, volumes of specific entities, such as FC or epiretinal proliferation (EPR) in eyes with LMHs, cannot be automatically extracted by current software. The need for complex processes to calculate volumes, requiring the use of deep learning machines or external software, significantly limits the practicality of utilizing volumetric analysis in clinical and surgical practice or the research field [1,6,17,18]. Indeed, although some machine learning-based tools have been introduced in the healthcare setting, prudence and vigilance are still recommended due to the persistence of various challenges [19]. Additionally, the use of external software requires extra procedures for installation and the exporting and uploading of OCT scans in the system for manual processing [1].

The aim of this study is to present and validate a novel and easy OCT-based method, without the need for deep learning machines or external software, that can manually calculate volumes of specific entities in retinal pathologies that cannot be automatically detected, segmented, or analysed by current OCT software. The method is accessible to anyone with an appropriate OCT instrument and may prove useful in both clinical and research settings for ophthalmologists in the evaluation and study of different pathologies.

## 2. Materials and Methods

**Figure 1:**
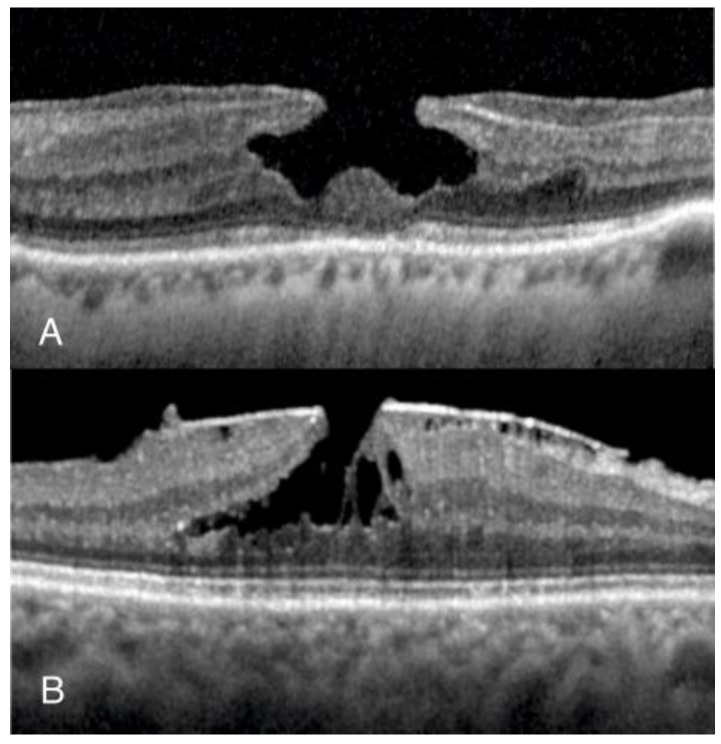
Sub-categories of lamellar macular holes. (**A**) Degenerative lamellar macular hole (D-LMH) is characterized by a foveal cavity with undermined edges and an irregular foveal contour with apparent loss of retinal tissue. (**B**) Epiretinal membrane foveoschisis (ERM-FS) is characterized by a contractile ERM with foveoschisis at the level of Henle-fibre layer.

A retrospective, non-interventional observational study was conducted, including eyes with a normal foveal profile (NFP) and LMH. Patients with NFP were identified by retrospectively evaluating the OCT scans of patients who attended Manchester Royal Eye Hospital for myodesopsias from January 2022 to January 2023 and eyes with a normal foveal profile were included in the study. Patients with LMHs were identified from the electronic surgical database of the vitreoretinal unit, which involved searching for those awaiting surgery from January 2020 to January 2023; both degenerative LMH (D-LMH) and ERM foveoschisis (ERM-FS), as defined by the OCT-based consensus definition for LMH [20] (Figure 1), were included. Exclusion criteria in both groups were (1) OCT scans with poor image quality; (2) myopia greater than 6 diopters; (3) severe glaucoma; (4) any concomitant retinal disease (other than LMH in the LMH group) involving the macula, such as retinal vein occlusion, age-related macular degeneration, diabetic maculopathy; (5) present or past history of uveitis; (6) history of trauma; and (7) previous intraocular surgery other than cataract surgery performed more than 6 months before vitrectomy.

The volume of the foveal area (defined as the retinal tissue within a circle of 500-micron radius centred on the foveal centre) was manually acquired in patients with NFP, and it was compared with the automated measurement provided by the OCT software; then, specific volumetric measurements that cannot be automated by current OCT software were measured in eyes with LMH. For both groups, only eyes with the “dense macular volume” preset of Heidelberg Spectralis (Engineering GmbH, Heidelberg, Germany) were included. This involves a macular volume scan acquisition with a 49-line horizontal raster and foveal centration, covering an area of 30° by 30°. Two observers (KS, NA) independently acquired the required measurements according to the method described below, and the inter-observer variability of data acquisition was evaluated. In addition, to evaluate the intra-observer variability, the same two observers independently re-acquired the same measurements six weeks later. In the event of outliers, a third observer (G.M.) conducted further review and checked the measurements acquired.

According to the United Kingdom (UK) guidance, retrospective data collection is regarded as an audit for service evaluation, and therefore ethical approval was not required.

### 2.1. Data Acquisition—General Protocol

The protocol for data acquisition includes the selection of each linear OCT scan that shows the presence of the specific entity of interest whose volume needs to be calculated. For each scan, the visualization mode was changed from the default vertical scaling (1:1 pixel) to 1:1 μm before the acquisition of data [21]. Using the freehand integrated calliper tool, the perimeter of the specific entity was drawn to acquire its desired cross-sectional area. The same process was repeated for all the scans. The distance between each linear scan was determined using the “Information” option on the OCT software. The volume of the specific entity of interest was finally calculated using the formula: Volume (mm^3^) = ∑area [mm^2^] × OCT-scan distance [mm].

### 2.2. Data Acquisition in the NFP Group

**Figure 2:**
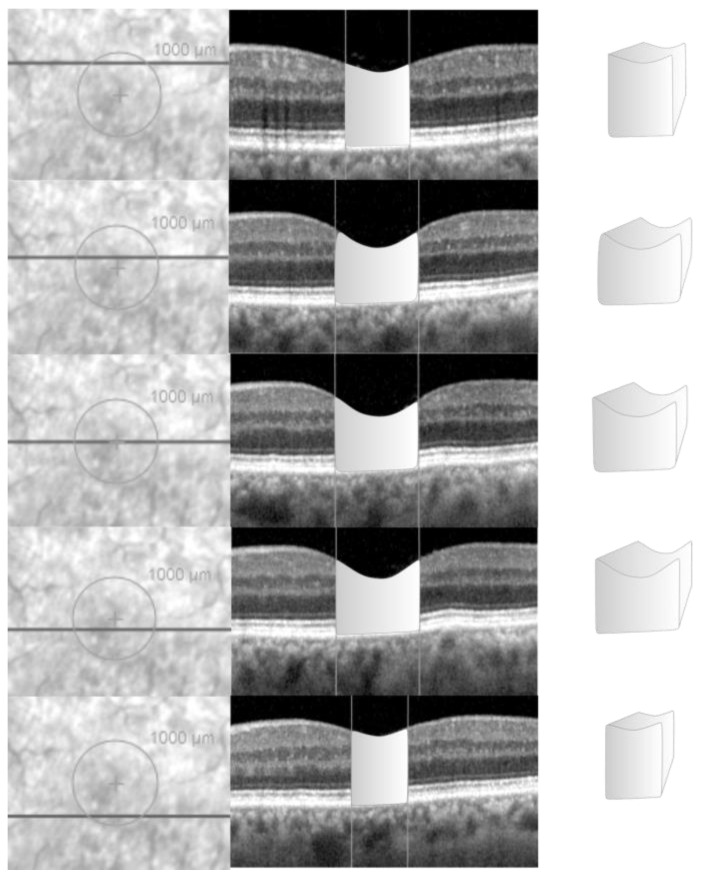
Calculation of retinal volume for normal foveal profile eyes. In order to calculate the volume of the retinal tissue in a circle of 1 mm diameter centred on the fovea centralis, on the infrared image, a circle—centred on the fovea and with a diameter of 1000 microns—was drawn using the calliper tool (first column). For each linear OCT scan within the circle, two vertical references were drawn at the nasal and temporal border of the circle (second column). The bi-dimensional area of retina within the circle was drawn and calculated by the machine using the calliper tool (second column). The area was multiplied for the distance between one scan and the following, calculating a small volumetric volume (third column). The total retinal volume, within the circle, was detected by summing all the single small volumetric volumes measured for each linear scan.

For each eye with NFP, the horizontal B-scan intersecting the centre of the fovea was identified and the vertical green reference line was adjusted to align with the foveal centre. This adjustment enabled the foveal centre to be highlighted on the infrared imaging (Figure 2).

A circle overlay, with a diameter of 1000 microns and centred on the fovea, was added to the infrared image in Spectralis. For each horizontal B-scan confined within the circle, the vertical green reference line was adjusted on the OCT scan to intersect the borders of the overlay, a vertical line was then drawn using the software tool on the OCT scan to highlight those borders as reference parameters. The perimeter of the retina, included between the two borders of the circle, was outlined to acquire its desired cross-sectional area (Figure 2). The same process described in the previous paragraph was used to calculate the volume of the retinal tissue, included within a diameter of 1000 microns across the fovea.

This manual volumetric volume was compared to the retinal volume within the central 1000 microns from the fovea, which was calculated automatically by Spectralis and extracted from the “Thickness Map” tab as the volume of the retina within the central circle of the 1, 3, and 6 ETDRS circle diameters.

### 2.3. Data Acquisition in the LMH Group

**Figure 3:**
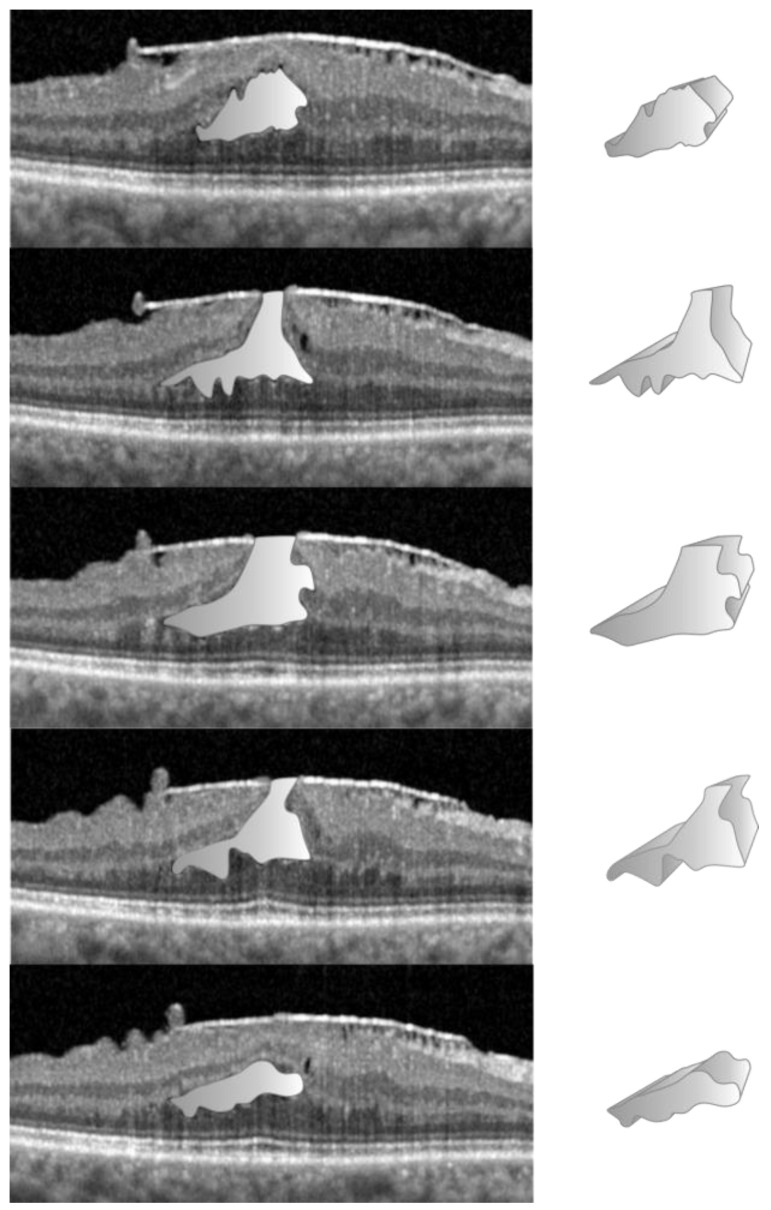
Calculation of foveoschisis volume for lamellar macular hole eyes. For each linear OCT scan, the border of the foveoschisis was delineated using the calliper tool (first column) and its area was calculated by the OCT software. The area was multiplied for the distance between one scan and the following, resulting in a small volumetric volume (second column). The sum of all the small volumes calculated for each linear scan resulted in the total volume of the foveoschisis.

The manual technique, to establish the volumes of specific entities like the FC in D-LMH or FS in ERM-FS, is more straightforward than NFP as no reference circle is required. From the “Display” tab, the aforementioned freehand calliper tool, present in the Spectralis system, was used to calculate the cross-sectional area of the FC or FS on a single horizontal B-Scan (Figure 3).

The FC/FS surface area was acquired for each linear OCT scan in which FC/FS was present and its volume was calculated using the previously described process. Due to the machine’s limitations in detecting and calculating these volumes, comparison with automated software measurements was not possible.

### 2.4. Statistical Analysis

All statistical analysis was performed using IBM SPSS Statistics for Windows, Version 29.0.2 (IBM Corp., Armonk, NY, USA). Statistical significance was defined as *p* < 0.05. Inter- and intra-observer variation and differences with automated OCT machine measurements were assessed with Bland–Altman plots [22]. The bias (mean of the differences in measurement) was presented with 95% confidence intervals (CIs) (± 1.96 times the standard error [SE] of the differences). The coefficient of repeatability (CR) was measured as the Standard Deviation [SD] of differences * 1.96. The 95% lower and upper limits of agreement (LOA) were calculated as bias ± CR. The 95% CIs of each respective upper and lower LOA were calculated as ±1.96 times the SE of the respective limit and approximated as per Bland et al. [SE = √((3s^2^)/*n*), where “s” is the SD of the differences and “*n*” is the sample size].

Power calculations for each comparison with Bland–Altman plots were performed as per Lu et al. [23]. The power calculation for a specified sample size was conducted using the following parameters: bias, SD of the bias, the targeted clinical agreement limit, available sample size, targeted upper and lower CIs (95% CI) for the LOAs and targeted confidence level of LOA (95%). The clinical agreement limit was determined as the maximum allowable difference that would be clinically significant, conservatively set to 0.01 mm^3^. Due to a sparsity in the literature regarding comparable measurements for NFP (volume measurements in the central 1 mm) and LMH groups, a power calculation was conducted after collecting 30 NFP and 32 LMH case pairs, respectively. Over 90% power was achieved, given the sample size in both groups.

## 3. Results

This study reports on 62 eyes, including 30 eyes with NFP and 32 with LMH. The former group was used to validate the proposed measurement method through the comparison of human and OCT automated measurements. In both groups, we assessed inter- and intra-observer variability.

**Figure 4:**
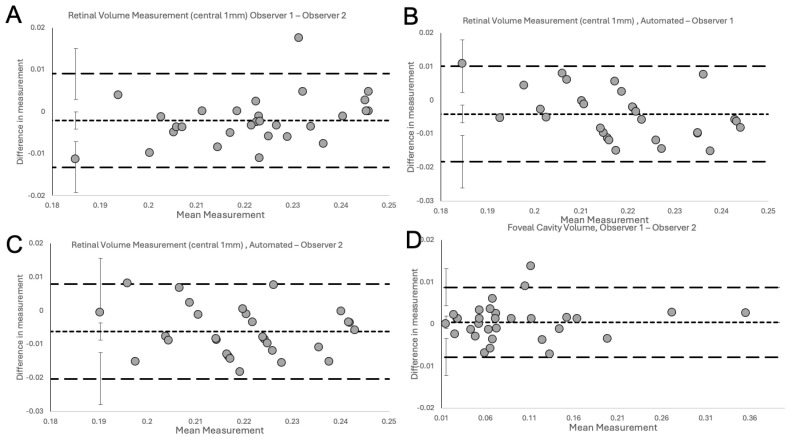
Inter-observer variation. The Bland–Altman plots delineate the degree of agreement for inter-observer variability between the two observers (**A**), observer 1 and Spectralis (**B**), observer 2 and Spectralis (**C**) for RV in the NFP group, and between the two independent observers for FC/FS volume in the LMH group (**D**). No systemic disagreement could be detected in all comparisons.

The Bland–Altman plots (Figure 4) show excellent agreement for inter-observer variability between the two observers (Figure 4A), between observer 1 and Spectralis (Figure 4B), between observer 2 and Spectralis (Figure 4C) for the measurements of RV in NFP eyes and between the two observers for measurements of the FC/FS volume in LMH eyes (Figure 4D).

The plots do not demonstrate systematic disagreement in all comparisons. Regarding RV measurements, both human observers had excellent agreement between themselves (Figure 4A, Table 1), and between Spectralis (Figure 4B,C, Table 1), with small mean differences and narrow LOA. Specifically, the small mean differences between human observers and Spectralis were negative (−0.0041 for observer 1 and −0.0062 for observer 2), showing that human observers had slightly larger measurements than the OCT software. The human observers had a single outlier in both a measurement in Figure 4A (RV) and Figure 4D (FC/FS), which, on revisiting by the third observer, were human errors in the measurement technique. With corrected measurements, these differences disappeared. However, we have presented the original measured data to avoid bias. The outlier in Figure 4A was a result of observer 1 slightly overestimating by error the area to be calculated, leading to larger-than-usual measurement. In contrast, observer 2, was slightly under-measuring this scan. It should be highlighted that the quality of the considered scan was slightly reduced, possibly leading to error. Nonetheless, both these errors were small, and both human measurements were within the LOA compared to the automated measurement (Figure 4B and 4C, respectively). In Figure 4D, observer 1 had not delineated an area of foveoschisis correctly, which led to overestimation of the volume of the schitic area.

**Figure 5:**
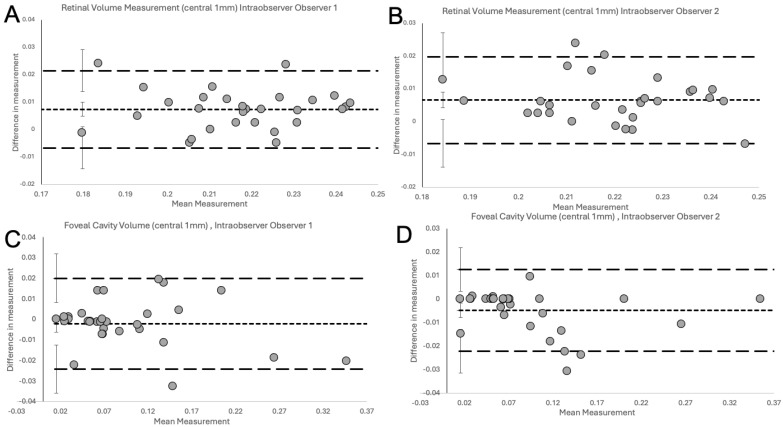
Intra-observer variation. The Bland–Altman plots delineate the degree of agreement for intra-observer variability between the baseline and repeated acquisitions between observer 1 (**A**) and 2 (**B**) for RV in the NFP group and between observer 1 (**C**) and 2 (**D**) for FC/FS volume in the LMH group. We found isolated outliers in intra-observer variability for observer 1 (**A**,**C**) and observer 2 (**B**,**D**); however, overall, no systemic disagreement could be detected in all comparisons.

After validation of the RV measurements, the agreement between both human observers for the calculation of specific volumes in pathological retinas, such as FC or FS volume measurements in eyes with LMH, was investigated. As Heidelberg Spectralis does not have an inbuilt facility to automate the volume measurement of FC/FS, the evaluation of agreement between observers and Spectralis was not possible for those specific volumes. Nonetheless, high agreement between both human observers using the novel volume measurement technique was demonstrated on the Bland–Altman plot (Figure 5D, Table 1). At the 6-week measurement, excellent intra-observer agreement with low variability and small differences in measurements were reported (Figure 5). These were within the 95% CI for the LOA and no systematic bias was observed.

## 4. Discussion

In both medical and surgical retinal pathologies, volumetric analysis has been proposed as an important tool to identify biomarkers for diagnosis, understanding the disease mechanisms, clinical grading, monitoring, and response to interventions [1,2,3,4,5,6,7,8]. For instance, in neovascular AMD, volumes of the intra- or sub-retinal fluid have been described as reliable biomarkers for disease activity as well as for visual function or outcomes, thus playing an important role in planning customized treatment strategies [5,9,10,11]. Similarly, in DR, evaluation of retinal or fluid cyst volumes allows for severity assessment or early detection of microvascular abnormalities, guiding interventions to prevent vision loss [3,6]. In multiple sclerosis optic neuritis, an increase in the inner nuclear layer (INL) volume has shown an association with the occurrence of clinical relapses [7]. In the glaucomatous field, 3D macular parameters (without the need for manual correction of artefacts in the clinical setting) have shown a diagnostic performance similar to or better than 2D measurements, allowing for the early diagnosis of glaucoma and prompt start of treatment, as well as valuable insights into the extent of optic nerve damage [24]. Recently, in the vitreoretinal field, Taşlıpınar Uzel et al. [1] highlighted the importance of volumetric analysis in D-LMH, reporting that FC volume was the only factor associated with baseline best corrected visual acuity (BCVA), whereas no correlation was detected between BCVA and linear OCT measurements, such as central retinal thickness [1]. Moreover, FC and ERP volumes were the only parameters that showed a significant increment at last follow-up visit compared to baseline, detecting changes in the natural course of D-LMH earlier than the horizontal diameter measurements [1]. In light of their findings, Taşlıpınar Uzel et al. [1] determined that tracking volumetric changes could offer deeper understanding of the dynamic transformations in retinal conditions like D-LMH, supporting physicians in making informed decisions regarding their treatment. 

Similarly, a recently published study demonstrated a highly predictive model for functional outcomes, following vitrectomy and internal limiting membrane (ILM) peeling in LMHs, utilizing specific OCT volumetric parameters, including FC or FS and ERP volumes [25]. However, unlike Taşlıpınar Uzel et al.’s [1] approach, which necessitated the use of external software, the method described herein did not require any external software to calculate the volume of specific entities, providing a simpler and quicker way to obtain volumes. Indeed, although ophthalmologists typically interpret retinal imaging using two-dimensional (2D) data subsets [2,26,27]; due to the increased interest in volumetric analysis, several OCT devices have enabled the segmentation of retinal layers in eyes with NFP, allowing for the automated measurement of their volume in the macular area [15,16]. However, these devices do not allow for automated calculation of specific entities in retinal pathologies. Therefore, to overcome this limitation, different strategies have been proposed in the scientific literature [1,6,17,18,28]. Among the strategies proposed for measuring the volume of specific entities in retinal pathologies, the processing of OCT scans using external software like ImageJ 2.0.0 (FIJI) was reported in the paper by Taşlıpınar Uzel et al. [1]. In their method, after exporting all linear OCT scans from the OCT software and uploading them into the external software, manual or semi-automated segmentation of the OCT images was required, using tools provided by the external platform, to trace the outlines of specific entities [1]. The use of external software platforms to calculate volumes requires several additional steps, such as installation, compatibility issues with different operating systems, dependencies, updates, additional costs, and the exportation of images. Moreover, they can have a steep learning curve for new users requiring significant commitment, particularly in users with limited experience in image analysis or programming. Finally, manual intervention, such as manual segmentation or the annotation of images—or human supervision in cases of semi-automated measurements—is required, while fully automated workflows can be challenging to implement, especially for complex image processing tasks or analyses requiring sophisticated algorithms. Beyond the use of external software, deep learning methods have emerged as powerful tools for the calculation of volumes, typically involving semantic segmentation techniques that classify each pixel in an image into predefined categories, thereby delineating different regions or structures of interest [6,17,18]. Starting from a large dataset of OCT images annotated with ground segmentations indicating the boundaries of the specific entities whose volumes need to be calculated, convolutional neural networks (CNNs) or other deep learning architectures such as U-Net, SegNet, and DeepLab architectures are chosen for semantic segmentation [29]. While our method can potentially offer more efficient data extraction for training supervised neural networks, several problems have been reported for deep learning models in the calculation of specific retinal entities, such as the inability of the model to segment small target regions [30], poor performance and challenges due to speckle noise and imaging artefacts [17], inaccurate measurements of fluid volume due to the sparse sampling density [18], the ability to segment exclusively entities with high contrast with surrounding tissues on OCT scans [6], and the insensitivity to the location of the target [6].

In contrast to other techniques, the proposed novel measurement method is applicable for any specific retinal entity of interest [25], using imaging protocols routinely present in a clinical setting. Indeed, in our method, the target areas for volumetric calculation are manually drawn using the inbuilt specific tool present in Heidelberg Spectralis from the display system. Moreover, if necessary, the novel technique described is applicable to both intra- and inter-retinal volumes. For instance, while in the LMH group the inter-retinal volume was acquired, in the NFP group the method was used to calculate the intra-retinal volume, requiring just a few additional steps. This method showed a high degree of agreement for the inter-observer variability for RV in NFP eyes and the FC/FS volume in LMH eyes, and between the single observers and the OCT software for RV in NFP eyes. Moreover, excellent intra-observer agreement for both the RV and FC/FS volumes was described. Compared with approaches based on external software, this method does not require exporting the OCT scans or uploading them to a different system, resulting in a significantly easier and less time-consuming procedure.

Limitations of this study include its limited sample size. However, these data were sufficient to highlight the excellent intra- and inter-observer agreement. Manual segmentation allows for detailed customization and fine-tuning of segmentation boundaries but can be time-consuming. Nonetheless, in small-sized studies with limited resources, manual acquisition may offer a more practical and cost-effective alternative, allowing researchers to maximize the utility of available resources without compromising on data quality or analysis.

In conclusion, an easy, reliable, and widely applicable method to calculate retinal volumes is described, showing high intra- and inter-observer agreement. In light of the clinical relevance of specific entities in several medical and surgical retinal diseases, we believe that this measurement method may be a valuable tool in both clinical and research settings.

## Figures and Tables

**Table 1:** Summary of 95% limits of agreement and coefficients of repeatability.

Type of Comparison	*n*	Estimate of Bias (mm)	95% CI (mm)	Lower Limit of Agreement (mm)	95% CI (mm)	Upper Limit of Agreement (mm)	95% CI (mm)	Coefficient of Repeatability (mm)
Intra-observer Total Retinal Volume								
Obs 1, Obs 1	30	0.0074	0.0048 to 0.0100	−0.0067	−0.0144 to 0.0010	0.0215	0.0138 to 0.0292	0.0141
Obs 2, Obs 2	30	0.0066	0.0042 to 0.0090	−0.0066	−0.0139 to 0.0006	0.0198	0.0125 to 0.0270	0.0132
Inter-observer Total Retinal Volume								
Obs 1-Obs 2	30	−0.0021	−0.0041 to −0.0001	−0.0132	−0.0193 to −0.0071	0.0090	0.0029 to 0.0151	0.0111
Automated-Obs 1	30	−0.0041	−0.0067 to −0.0015	−0.0184	−0.0262 to −0.0106	0.0102	0.0024 to 0.0180	0.0143
Automated-Obs 2	30	−0.0062	−0.0088 to −0.0036	−0.0203	−0.0280 to −0.0126	0.0079	0.0002 to 0.0156	0.0141
Intra-observer Foveal Cavity								
Obs 1, Obs 1	32	−0.0021	−0.0061 to 0.0020	−0.0242	−0.0359 to −0.0125	0.0201	0.0083 to 0.0318	0.0221
Obs 2, Obs 2	32	−0.0048	−0.0080 to −0.0016	−0.0222	−0.0315 to −0.0130	0.0126	0.0034 to 0.0219	0.0174
Inter-observer Foveal Cavity								
Obs 1, Obs 2	32	−0.0004	−0.0020 to 0.0011	−0.0088	−0.0132 to −0.0043	0.0079	0.0035 to 0.0123	0.0083

The table shows the 95% limits of agreement and coefficients of repeatability for intra-observer and inter-observer analysis (each respective observer and Heidelberg Spectralis automated measurements) for retinal volume in the central 1 mm and the foveal cavity or foveoschisis in eyes with lamellar macular holes.

## Data Availability

No new data were created for this article.
